# Domain-swapping of mesophilic xylanase with hyper-thermophilic glucanase

**DOI:** 10.1186/1472-6750-12-28

**Published:** 2012-06-07

**Authors:** Liangwei Liu, Linmin Wang, Zhang Zhang, Xiaodan Guo, Xiangqian Li, Hongge Chen

**Affiliations:** 1 Life Science College, Henan Agricultural University, 95 Wenhua Road, Zhengzhou, Henan, 450002, China; 2 School of Life Science, Huaiyin Institute of Technology, 3 Meicheng Road, Huaian, Jiangsu, 223001, China; 3 Key Laboratory of Enzyme Engineering of Agricultural Microbiology, 95 Wenhua Road, Zhengzhou, Henan, 450002, China

**Keywords:** Xylanase, Glucanase, Domain-swapping, Fusing

## Abstract

**Background:**

Domain fusion is limited at enzyme one terminus. The issue was explored by swapping a mesophilic *Aspergillus niger* GH11 xylanase (Xyn) with a hyper-thermophilic *Thermotoga maritima* glucanase (Glu) to construct two chimeras, Xyn-Glu and Glu-Xyn, with an intention to create thermostable xylanase containing glucanase activity.

**Results:**

When expressed in *E. coli* BL21(DE3), the two chimeras exhibited bi-functional activities of xylanase and glucanase. The Xyn-Glu Xyn moiety had optimal reaction temperature (T_opt_) at 50 °C and thermal in-activation half-life (t_1/2_) at 50 °C for 47.6 min, compared to 47 °C and 17.6 min for the Xyn. The Glu-Xyn Xyn moiety had equivalent T_opt_ to and shorter t_1/2_ (5.2 min) than the Xyn. Both chimera Glu moieties were more thermostable than the Glu, and the three enzyme T_opt_ values were higher than 96 °C. The Glu-Xyn Glu moiety optimal pH was 5.8, compared to 3.8 for the Xyn-Glu Glu moiety and the Glu. Both chimera two moieties cooperated with each other in degrading substrates.

**Conclusions:**

Domain-swapping created different effects on each moiety properties. Fusing the Glu domain at C-terminus increased the xylanase thermostability, but fusing the Glu domain at N-terminus decreased the xylanase thermostability. Fusing the Xyn domain at either terminus increased the glucanase thermostability, and fusing the Xyn domain at C-terminus shifted the glucanase pH property 2 units higher towards alkaline environments. Fusing a domain at C-terminus contributes more to enzyme catalytic activity; whereas, fusing a bigger domain at N-terminus disturbs enzyme substrate binding affinity.

## Background

Enzyme is important in biomass conversion and renewable energy production. However, biotechnological condition demands for thermostable enzymes. Thermostability can be enhanced by fusing proper protein fragments, such as, the non-homologous fragments [1], the thermophilic xylanase homologous N-terminus [2,3], the hyper-thermophilic carbohydrate binding-module [4], and the *Pseudoalteromonas arctica* esterase OsmC domain [5]. Two catalytic domains, such as, xylanase and glucanase, can also be combined to create bi-functional chimeras [6-8]. However, the domain fusions were limited at xylanase one terminus. When fused at the other terminus, the *Clostridium thermocellum* xylanase and *Thermotoga maritima* glucanase activities were damaged [7-9]. Thereby, domains need to be swapped with each other in fusion investigation.

**Figure 1:**
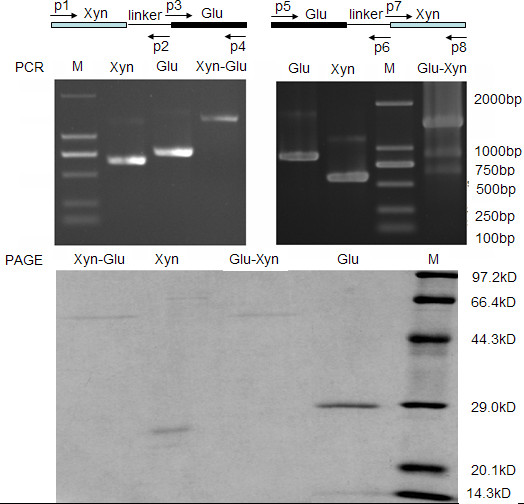
** Construction of the chimeras.** The domains Xyn and Glu were connected by overlapping-extension PCR. The genes *xyn* and *glu* were amplified using p1/p2 and p3/p4, respectively, and the *xyn-glu* was created using p1/p4 (Upper). Thereafter, the genes *glu* and *xyn* were amplified using p5/p6 and p7/p8, respectively. The *glu-xyn* was created using p5/p8. The wild Xyn and Glu were amplified using p1/p8 and p5/p4, respectively. The fusion genes appear on the gel as discrete bands at ~ 1.4 kb, with molecular masses of approximately the sum of the contributing gene fragments (Middle). The two chimeras appear on the SDS-PAGE gel as discrete bands at ~ 53 kDa, with molecular masses of approximately the sum of the Xyn, linker peptide, and Glu (Lower). Linker: linker-peptide, M: marker of DNA and protein.

Xylanase (EC3.2.1.8) is widely used in feed, flour, baking industry, pulp bleaching, etc [3,10,11]. An *Aspergillus niger* xylanase (Xyn) has high catalytic activity (GenBank: EU375728) [12]. The 185 residue enzyme is more suitable for domain fusion investigation, because it is the smallest xylanase in GH11 family. However, the Xyn optimal temperature for activity (T_opt_) is 47 °C, and its thermal inactivation half-life (t_1/2_) at 50 °C is 17.6 min [4]. To break down complicated biomass [13], the mesophilic fungal Xyn needs enhanced thermostability and synergistic glucanase activity. An ideal partner is the *T. maritima* glucanase (Glu) (EC3.2.1.4), because it is hyper-thermophilic and 258 residue big (Met_1_-Glu_258_) [14,15]. Linker-peptide is also important for enzyme thermostability [16,17], because each domain needs necessary space to form to active conformation [4]. A linker-peptide (Pro_692_-Gly_713_), which was selected from the hyper-thermophilic *T. maritima* xylanase A [10], was confirmed having enough space for connecting two domains [4]. Thereby, the 22 residue linker-peptide is used to connect the *A. niger* Xyn with the *T. maritima* Glu to construct two chimeras, Xyn-Glu and Glu-Xyn (Figure 1). After expression in *E. coli* BL21(DE3), the two chimeras exhibit bi-functional activities of xylanase and glucanase. Especially, fusing the Glu domain at C-terminus increases the mesophilic xylanase T_opt_ value and thermostability. The domain-swapping provides more insights into enzyme fusion investigation.

## Results and discussion

### Construction of chimeras

After step-extension PCR, the *xyn-glu* and *glu-xyn* construct genes appeared on the 1.4 % electrophoresis gel as discrete DNA bands at ~1.4 kb, approximately the combined molecular masses of contributing gene fragments (Figure 1). After the sequence accuracies had been confirmed for the recombinant plasmids (GenBank: JQ793634, JQ793635), transformed cells containing pET20b-*xyn-glu* and pET20b-*glu*-*xyn* were induced to express the chimeric xylanases. The two proteins, Xyn-Glu and Glu-Xyn, appeared on the 10 % SDS-PAGE gel as discrete bands at ~ 53 kDa. As expected, the molecular masses are approximately the sum of two contributing moieties (Figure 1, Table 1), because the chimeras have residues equaling to the sum of Xyn, Glu, and linker peptide.

**Table 1:** Enzyme property of the chimeras

**Enzyme**	**Xyn Activity**			**Glu Activity**			**Physical feature**	
	**t**_1/2_**-50 °C (min)**	**pH**_opt_/**T**_opt_**(°C)**	**K**_m_**(mg/ml)/K**_cat_**(s**^-1^**)**	**t**_1/2_**-95 °C (min)**	**pH**_opt_**/T**_opt_**(°C)**	**K**_m_**(mg/ml)/K**_cat_**(s**^-1^**)**	**MM(kDa) Apparent/Theoretical**	**Number of residues/pI**
Xyn-Glu	47.6	3.8/50	10.2/118.9	> 99.8	3.8/≥ 96	5.9/3.4	53/ 53.2	465/4.5
Glu-Xyn	5.2	3.8/47	44.2/50.2	99.8	5.8/≥ 96	1.88/15.9	53/53.2	467/4.5
Xyn	17.6	3.8/47	12.3/49.2				25/ 21.1	185/4.5
Glu				96.2	3.8/≥ 96	1.87/2.6	29/ 30.9	258/4.78

pH_opt_: optimal reaction pH, T_opt_: optimal reaction temperature, t_1/2_ (min): thermal inactivation half-life, the residual xylanase activity was assayed after incubation at 50 °C at 10-min intervals from 0 – 30 min, and the residual glucanase activity was assayed after incubation at 95 °C at 20-min intervals from 0 – 60 min. K_m_ and K_cat_ were determined for birch-wood xylan and carboxymethyl cellulose sodium (CMC).

### Properties of the chimeras

**Figure 2:**
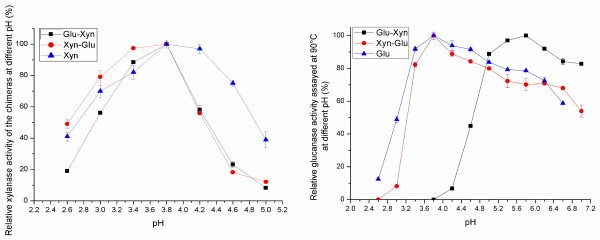
** Optimal pH of both moieties.** The Glu-Xyn Xyn moiety (■) has equivalent pH_opt_ to the Xyn-Glu Xyn moiety (●) and the Xyn (▲) (Left). The Glu-Xyn Glu moiety has pH_opt_ at 5.8, 2 units higher than the Xyn-Glu Glu moiety and the Glu (▲) (Right).

**Figure 3:**
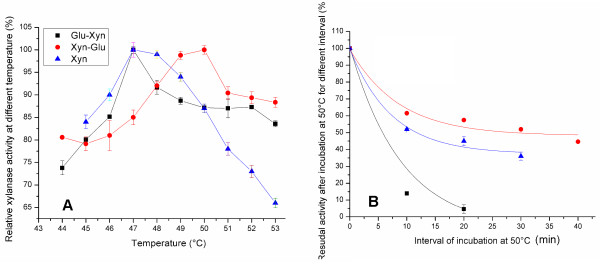
** Thermal property of Xyn moiety.** The Xyn-Glu Xyn moiety (●) has 3 °C higher T_opt_ than the Glu-Xyn Xyn moiety (■), and its T_opt_ equals to that of the Xyn (▲) (A). The residual xylanase activity was assayed after incubation at 50 °C at 10-min intervals from 0 – 30 min (B). The Xyn-Glu Xyn moiety t_1/2_ was 47.6 min, 2.7-fold longer than the Glu-Xyn Xyn moiety (5.2 min) and the Xyn (17.6 min).

When properties were assayed, the two chimeras exhibited bi-functional activities of xylanase and glucanase. When xylanase activity was determined, both chimera Xyn moieties had optimal reaction pH (pH_opt_) at 3.8, equivalent to the Xyn. Thus, fusing the Glu domain at either terminus did not alter the xylanase pH property. Both chimera Xyn moiety activities decrease at pH higher than 3.8 (Figure 2). As to thermal property, the Xyn-Glu Xyn moiety had optimal reaction temperature, T_opt_, at 50 °C, compared to 47 °C for the Xyn (Figure 3A). The Xyn moiety had t_1/2_ at 50 °C for 47.6 min, ~ 2.7-times longer than the Xyn (Figure 3B, Table 1). Fusing the Glu domain at C-terminus increased the xylanase thermo-activity and thermostability. In contrast, the Glu-Xyn Xyn moiety had T_opt_ at 47 °C, equivalent to the Xyn (Figure 3A). However, the Xyn moiety had t_1/2_ value at 50 °C for 5.2 min, shorter than the wild Xyn. Fusing the Glu domain at N-terminus did not alter the xylanase thermal activity, but decreased its thermostability (Figure 3B, Table 1).

**Figure 4:**
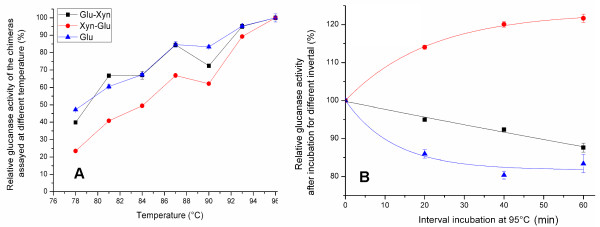
** Thermal property of Glu moiety.** Both chimera Glu moiety T_opt_ values were higher than 96 °C, similar to the wild Glu (▲) (A). The residual glucanase activity was assayed after incubation at 95 °C at 20-min intervals from 0 – 60 min (B). The Glu-Xyn Glu moiety (■) t_1/2_ was 99.8 min, shorter than the Xyn-Glu Glu moiety (●) and longer than the Glu (96.2 min).

When glucanase activity was determined, both chimera Glu moiety T_opt_ were higher than 96 °C, similar to the Glu (Figure 4A). The Glu-Xyn Glu moiety had t_1/2_ at 95 °C for 99.8 min, and the Xyn-Glu Glu moiety had t_1/2_ clearly longer than 99.8 min (Figure 4B, Table 1). The Glu activity increased after incubation at 95 °C, because the chimera T_opt_ was higher than 96 °C and its thermostability was significantly high. Fusing the Xyn domain at either terminus, especially, at N-terminus, increased the glucanase thermostability. The Glu-Xyn Glu moiety pH_opt_ was 5.8, 2 units higher than the Xyn-Glu Glu moiety and the wild Glu (Figure 2). Fusing the Xyn domain at C-terminus significantly shifted the glucanase pH property to higher pH environments.

When the kinetics were determined, the xylanase catalytic efficiencies (K_cat_) toward birch-wood xylan increased in the order Xyn < Glu-Xyn < Xyn-Glu. The substrate-binding affinities (K_m_) for birch-wood xylan increased in the order Glu-Xyn < Xyn < Xyn-Glu (Table 1). Fusing the Glu domain at either terminus cooperated with the xylanase to degrade substrate; however, fusing the Glu domain at N-terminus interfered with the xylanase substrate-binding affinity. In contrast, the glucanase catalytic efficiencies (K_cat_) toward carboxymethyl cellulose sodium (CMC) increased in the order Glu < Xyn-Glu < Glu-Xyn. The substrate-binding affinities (K_m_) for CMC increased in the order Xyn-Glu < Glu = Glu-Xyn (Table 1). Fusing the Xyn domain at either terminus cooperated with the glucanase to degrade substrate; however, fusing the Xyn domain at N-terminus interfered with the glucanase substrate-binding affinity. Thus, a domain, especially, a bigger one, is better fused at C-terminus for enzyme getting higher activity.

The Xyn-Glu Xyn moiety T_opt_ was 3 °C higher than the Xyn. This is consistent with the Xyn T_opt_ 3 °C increase after fusing the hyper-thermophilic carbohydrate-binding module [4]. Previous domains were mainly fused at enzyme one terminus. For example, the *Bacillus subtilis* xylanase was fused at the *Bacillus amyloliquefaciens* glucanase C-terminus [6]. The *T. maritima* xylanase was fused at the *T. maritima* glucanase C-terminus [8]. The *T. maritima* β-glucosidase was fused at the *T. maritima* cellulase C-terminus [9]. When the domains were fused at the other terminus, the chimeras lost both moiety activities [6,8]. There are so many xylanase domains fused at glucanase C-terminus. The situation probably indicates that xylanase locates naturally at downstream of glucanase. It is probably a trace of natural evolution, because the site location order is shown in the glucanase-xylanase bi-functional protein selected from meta-genome library [13]. The domain-swapping created different effects on each moiety properties. The N-terminal Glu domain decreased the xylanase thermostability; however, the C-terminal Glu domain increased the xylanase thermostability. The Glu has 258 residues, ~ 1.4-times bigger than the 185 residue Xyn. Probably, the C-terminal Glu protects the Xyn from thermal inactivation; whereas, the N-terminal Glu interferes with the Xyn folding and confirmation-forming, therefore, interferes with its thermostability. In contrast, fusing the Xyn at either terminus, especially, at N-terminus, increased the glucanase thermostability. In addition, fusing the Xyn at C-terminus shifted the glucanase pH_opt_ 2 units higher to alkaline environment. The result probably reflects that glucanase cooperates with other catalytic domains on both sides, because two glucanases, CelA and CelB, are shown connecting together in the *T. maritima* genome (EMBL-Bank: Z69341.1) [14].

Each domain has a natural conformation to perform catalytic activity. Therefore, each domain needs a necessary space to form to an active conformation [4,17-19], that is the reason why a suitable linker-peptide is so important. The decreased chimera T_opt_ values in previous investigations are attributed to linker-peptide interference. For example, fusing the *B. amyloliquefaciens* β-glucanase decreased the *B. subtilis* xylanase T_opt_ value and thermostability [6]. Fusing the *T. maritima* glucanase also decreased the xylanase T_opt_ value. When the domains were fused at other terminus, many enzyme activities were damaged [6,8]. The phenomenon is attributed to lack of linker-peptide. For example, the glucanases lost activities when fused at the other terminus [8,9]. To exclude the linker-peptide disturbance, we used the natural 22 residue linker-peptide to connect the Glu and Xyn. In addition, boundary determination is important for each domain to have complete residues. Only a domain has complete residues, can it form to an active confirmation. In the meantime, we have to eliminate the extra residues encoded by unnecessary endo-nuclease sites in expression vectors. Otherwise, the extra residues would disturb enzyme properties [20]. Thereby, *Nde*I and *Xho*I were used to delete the unnecessary endo-nuclease sites within the pET20b(+).

## Conclusions

Through the natural 22 residue linker-peptide, the mesophilic *A. niger* Xyn was fused and swapped with the hyper-thermophilic *T. maritima* Glu. After expression in *E. coli*, both chimeras exhibited bi-functional activities of xylanase and glucanase. Especially, the Xyn-Glu Xyn moiety had 3 °C higher T_opt_ and 2.7-times higher thermostability than the Xyn. In addition to the synergistic glucanase activity, the fungal xylanase was enhanced thermostability. The domain-swapping created different effects on each moiety. Fusing the Glu domain at C-terminus increased the xylanase thermostability, but fusing the Glu domain at N-terminus decreased its thermostability. Fusing the Xyn domain at either terminus increased the glucanase thermostabilities, and fusing the Xyn domain at C-terminus shifted the glucanase pH property 2 units higher towards alkaline environments. From the domain-swapping investigation, we can infer that a domain, especially a larger one, can only be fused at C-terminus to increase enzyme catalytic efficiency.

## Methods

### Materials and reagents

The *A. niger* Xyn gene (GenBank: EU375728), which encodes a 185 residue (M_1_-S_185_) mature xylanase, was cloned into pET20b(+) (Novagen, Shanghai, China). The *T. maritima* Glu gene (GenBank: Z69341), which encodes a 258 residue (M_1_-E_258_) mature glucanase, was cloned into pET20b(+) [15]. The 22 residue linker-peptide (P_692_-G_713_), which was selected from the *T. maritima* xylanase A (GenBank: Z46264), was used to connect the two domains [4] (Figure 1). Molecular reagents including *Pfu* polymerase, *Nde*I and *Xho*I, T4 DNA ligase, and DNA and protein marker were purchased from Takara Inc (Dalian, China).

### Construction of chimeras

Through the linker-peptide, the two domains were fused together by overlapping-extension PCR. The standard PCR was carried out using 16.5 μg of pET20b-*xyn* or pET20b-*glu*, 1.0 μl of each of two related primers, 5 U of *Pfu* polymerase, 4.0 μmol of dNTPs, and 1 × polymerase buffer with the following thermal cycling: 4 min denaturation at 95 °C, followed by 30 cycles of 1 min denaturation at 94 °C, 1 min annealing at 33 °C, and 1 min extension at 72 °C. The reaction was completed with a 10 min extension at 72 °C, unless described otherwise.

The genes *xyn* and *glu* were amplified using p1/p2 and p3/p4 and annealing at 24 °C (Figure 1). The genes were recovered and served as templates (each 11 μg) to amplify the *xyn-glu* using p1/p4, annealing at 62.4 °C, and extending for 1.5 min. The genes *glu* and *xyn* were amplified using p5/p6 and p7/p8, and annealing at 36 °C. The amplified genes were recovered and served as templates (each 11 μg) to amplify the *glu-xyn* using p5/p8, annealing at 65 °C, and extending for 1.5 min. The wild Xyn and Glu were amplified using p1/p8 and p5/p4, respectively. The primers were shown in Table 2, with italic shown for *Nde*I/*Xho*I restriction sites and bold for homologous region.

**Table 2:** Primers used to construct chimeras

**primer**	**sequence**
p1	GGAATTC***CATATG***AGTGCCGGTATC
p2	**CCAACGCTCGTCAGGTACGAGTC**
p3	**GACTCGTACCTGACGAGCGTTGG**
p4	CC**AA**ATTA***CTCGAG***AACTTCGACAGAG
p5	GAATTC***CATATG***ACGAGCGTTGG
p6	**GTTGATACCGGCAGTCAGGTACGAGTCATCC**
p7	**GGATGACTCGTACCTGAGTGCCGGTATCAAC**
p8	ATTA***CTCGAG***AGAGGAGATCGTGAC

Following PCR amplification, the genes were cloned into pET20b(+) plasmids that had been digested with *Nde*I/*Xho*I to delete the redundant endo-nuclease sites. The recombinant plasmids were transformed *E. coli* BL21(DE3) competent cells, then extracted and sequenced with an ABI 3730 automated DNA sequencer to confirm gene accuracy (Invitrogen Biotechnology, Shanghai, China). Accurately transformed plasmids were grown and induced to produce enzymes according to standard protocols [4]. A C-terminal His_6_ tag was included in the chimera sequences to allow the proteins to be purified with Co^2+^-binding resin (Amersham Bioscience). Active fractions were pooled and further purified using sephadex G-25. The enzymes were detected using 12 % polyacrylamide SDS-PAGE, stained with Coomassie brilliant G-250. Protein concentrations were measured according to the Lowry method.

### Enzyme properties of the chimeras

Each moiety of both chimeras was assayed in parallel with the Xyn or Glu, respectively. Every data point was determined for three independent reactions, including protein concentration, substrate concentration, optimal reaction temperature (T_opt_), optimal reaction pH (pH_opt_), residual activity, etc. The pH_opt_ value was determined from pH 2.6 – 5.0 in imidazole-biphthalate buffers. The Xyn moiety T_opt_ was determined from 44 – 53 °C, and residual activity was assayed after incubation at 50 °C at 10-min intervals from 0 – 40 min. The Glu moiety T_opt_ was determined from 78 to 96 °C, and residual activity was assayed after incubation at 95 °C at 20-min intervals from 0 – 60 min. To indicate thermostability, residual activity was expressed as a ratio relative to the un-incubated enzyme activity, and the thermal in-activation half-life (t_1/2_) was calculated by fitting the data with the equation y = A^*^e^*-kt*^ (Origin, version 8.0).

The kinetics were assayed at T_opt_ and pH_opt_ conditions using birch wood xylans at concentrations from 10 – 40 mg/ml for 5 min (Sigma-Aldrich, Shanghai, China). The kinetics for glucanase were assayed at 85 °C and pH_opt_ conditions using CMC at concentrations from 2.5 – 15 mg/ml for 5 min. The data were fitted with the Hill function to calculate maximal activity (V_max_) and K_m_ (Origin, version 8.0). Xylanase activity was determined toward birch-wood xylan and glucanase activity was determined toward CMC using the dinitrosalicylic acid (DNS) method described previously [4]. One unit of activity (U) was defined as the amount of enzyme that produced 1 μmol xylose or glucose per minute.

## Competing interests

The authors declare that they have no competing interests.

## Author’s contributions

Liu, L. conceived of the study and participated in its design. Wang, L., Zhang, Z., and Guo, X. carried out the molecular genetic studies. Li, X. and Chen, H. performed the statistical analysis. All authors read and approved the final manuscript.
